# Pseudoangiomatous stromal hyperplasia: an unsuspected cause of anisomasty

**DOI:** 10.1080/23320885.2020.1824614

**Published:** 2020-10-06

**Authors:** Fabio Santanelli di Pompeo, Michail Sorotos, Francesca Passarelli, Valeria Berrino, Guido Firmani, Harm Winters, Guido Paolini

**Affiliations:** a Plastic Surgery Unit, Sant'Andrea Hospital, School of Medicine and Psychology, ‘Sapienza’ University of Rome, Rome, Italy; b Department of Medicine, Surgery and Dentistry ‘Scuola Medica Salernitana’, PhD School of Translational Medicine of Development and Active Aging, Università degli Studi di Salerno, Salerno, Italy; c Pathology Unit, IDI‐IRCCS, Rome, Italy; d Department of Plastic, Reconstructive and Hand Surgery, Radboud University Nijmegen Medical Centre, Nijmegen, The Netherlands

**Keywords:** Breast asymmetry, Pseudoangiomatous stromal hyperplasia, PASH, benign tumour

## Abstract

Breast asymmetry can be congenital or developmental, however a tumorous growth may be the cause of this condition after puberty. A 19-year-old female presented with a slowly developing breast asymmetry pre-operatively diagnosed as Pseudoangiomatous Stromal Hyperplasia (PASH). The patient underwent tumour excision with breast gland remodelling. Postoperative course was uneventful.

## Introduction

Breast asymmetry is characterised by differences in the size, shape or position of the breasts [1]. It may cause psychological and emotional concerns and can be a reason for patients to consult a plastic and reconstructive surgeon. Its etiopathogenesis can be congenital (e.g. Poland's Syndrome) or developmental (e.g. tuberous breasts) [2]. In 1984, Van Den Bussche et al. proposed a classification system consisting of four main groups: (1) True malformation asymmetry, in which deformities of the breast, the pectoral muscles or the thoracic wall is present; (2) Precocious asymmetry, which starts at puberty with asymmetrical breast development and no previous anomaly; (3) Secondary or progressive acquired breast asymmetry, a slowly acquired asymmetry most often after pregnancy; (4) Tertiary or induced breast asymmetry which is the result of trauma or surgical treatment. According to the classification system, slowly growing masses may cause type 3 secondary or progressive acquired breast asymmetry. Regarding tumours, phyllodes tumour and lipomas are most likely to lead to a noticeable volume change in a woman’s breast [3–5]. However, when the asymmetry becomes apparent during or after puberty it may be misdiagnosed with type 2 precocious or developmental asymmetry. In type 2 asymmetries, the breast morphology is examined to determine whether the problem is unilateral or bilateral. An essential aspect is understanding what the patient perceives as abnormal. In some cases, most often in asymmetries of volume, it is possible to operate on one breast with a breast reduction alone or lipofilling in the smaller breast, avoiding implants. Differential reductions, mastopexies, augmentations, and most frequently combinations of these achieve the most harmonious balance between the breasts, especially in asymmetries of shape. In type 3 asymmetries, breast reconstruction depends on the oncological procedure and location of the mass: when mastectomy is not indicated, breast symmetry can be achieved with lipofilling, Wise pattern or Modified Wise pattern quadrantectomy [6].

## Case report

A 19-year-old female reported breast asymmetry slowly developing since the beginning of breast development at the age of 13. During a 6-year period, the left breast gradually increased in size and was misdiagnosed as a developmental asymmetry by her paediatrician. Besides aesthetic and emotional problems, the patient had no pain in the breasts or showed any discharge from the nipple.

**Figure 1.**
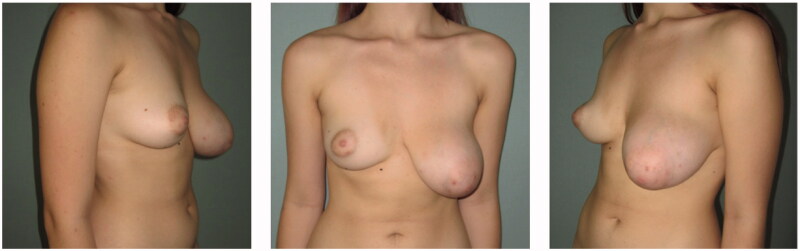
Preoperative frontal (center), left oblique (left side), right oblique (right side) pictures.

Upon physical inspection, the left breast appeared larger but with symmetrical inframammary folds (IMF) and there were no signs of a breast malignancy such as dermal pitting, flaking or erythema, or presence of a sunken or inverted nipple (Figure 1).

Left vs right breast measurements were: Sternal to nipple distance 27.5 vs 21 cm, IMF to nipple distance 12 vs 9.5 cm, NAC diameters 7 vs 4 cm. Breast volume was calculated with the BREAST-V application, was 693 cc for the left and 336 cc for the right breast [7].

No palpable masses were identified on the right breast, while on the left breast a large firm mass was presently occupying all quadrants. Endocrinology consultation was negative since blood analysis revealed prolactin levels 24.8 ng/ml at time 0, 21.3 ng/ml at 15′ and 18.3 ng/ml at 30′ (range 2–29 ng/ml), TSH was 1.670 μIU/ml (range 0.4–4 μIU/ml), growth hormone 0.31 ng/ml (1–14 ng/ml) and the patient had a normal menstruation cycle. Ultrasound showed a solid nodular mass of at least 10 cm diameter with compacted glandular characteristics, contrast-enhanced MRI imaging confirmed a coarse formation of 90 × 70 mm, capsulated, with uneven uptake after contrast enhancer, likely a benign lesion, BRADS 3. Core needle biopsy (CNB) was carried out and resulted in the diagnosis of Pseudoangiomatous Stromal Hyperplasia (PASH).

**Figure 2.**
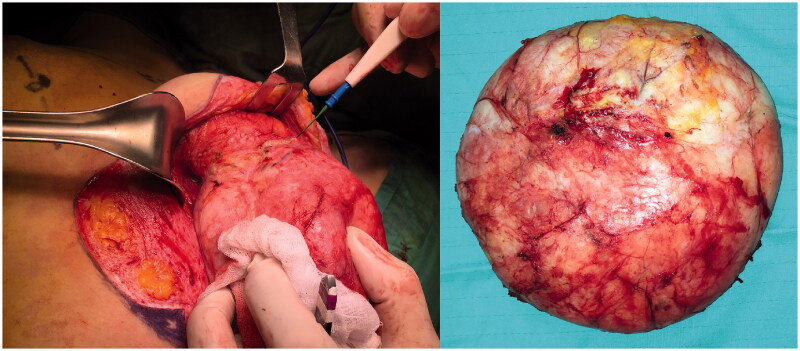
Intraoperative view showing well-encapsulated tumorous growth with a distinct dissection plane (left). Photo of tumorous growth upon removal (right).

**Figure 3.**
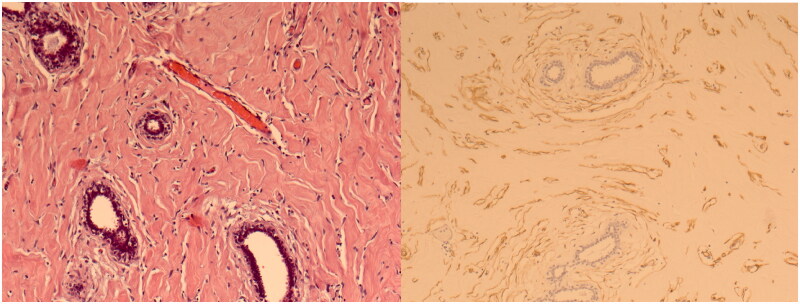
Left side: Proliferation of stromal elements (fibroblastic/myofibroblastic) mixed with breast ducts (10× HE). Right side: Dense keloid-like stroma has anastomosing pattern of slit-like clefts (empty spaces) lined by single layer of flat spindle cells simulating vascular spaces CD34 + (10× CD34).

**Figure 4.**
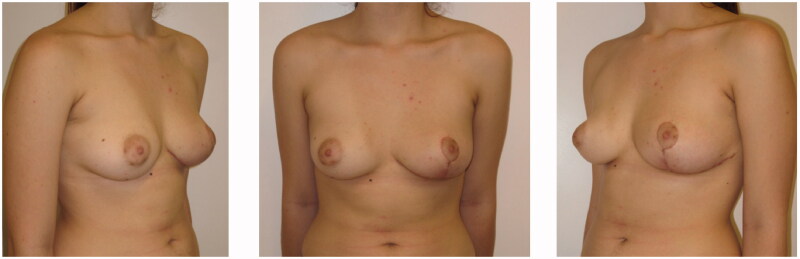
Postoperative frontal (center), left oblique (left side), right oblique (right side) pictures.

Under general anaesthesia, the patient underwent removal of the tumour with remodelling of the left breast by means of a Wise pattern skin reduction approach. The tumorous mass appeared well encapsulated and was easily dissected from the surrounding tissues (Figure 2). It was sent for definitive pathology that reported mammary tissue with stromal myofibroblastic proliferation characterized by pseudovascular slit spaces connected to each other and coated by splindle cells, free from atypia, confirming the PASH diagnosis (Figure 3). After day 1 the patient was discharged, and recovered uneventful and she was satisfied with the final results. At 12 months follow-up, there were no signs of recurrence (Figure 4).

## Discussion

PASH, first described by Vuitch et al. [8], is a condition characterized by hyperplasia of stromal myofibroblasts in response to hormonal stimuli. The tissue strongly resembles an angiomatous proliferation upon histological analysis, hence the term ‘pseudoangiomatous’. It is a benign tumour with similar clinic and radiologic characteristics to fibroadenoma [9–11]. The histological features may lead to the misdiagnosis of low-grade angiosarcoma, but the channel found in PASH are not true vascular spaces but slit-like spaces lined by myofibroblasts caused by disruption and separation of stromal collagen fibres. In these cases, immunochemistry is helpful [12].

In most cases, PASH is an incidental histologic finding, although considered a rare condition, its prevalence is not well defined due to conflicting literature reports. Ibrahim et al. [13] demonstrated that 23% out of 200 histologic samples excised for various malignant and benign reasons contained PASH. However, in a study by Polger et al. [14] PASH was identified in 0.4 percent out of 1661 samples. Early reports most commonly described PASH in pre-menopausal women or in elder women taking estrogenic replacement therapy. It is however also described in men with gynecomastia and in immune-suppressed patients. It may also present as a mass or nodule and it is typically solitary, circumscribed, rubbery and mobile; macroscopically it presents as a well-circumscribed fibrous mass which can be white, grey or tan in colour [12,14]. Because it can sometimes rapidly increase in size, PASH may be causing concern regarding a possible malignant nature of the tumour.

In this case, the PASH was of such size that it caused symptomatic breast asymmetry which was initially misdiagnosed as developmental breast asymmetry. This illustrates that a clinician should be aware that breast asymmetry might be caused by more uncommon conditions even when initial patient history and anamnesis would suggest otherwise. Therefore, in these patients, careful clinical examination and radiological examinations should always be performed for a correct diagnosis and preoperative planning that leads to tumour removal and achievement of aesthetically pleasant symmetrical results.
